# Comparative genomic analysis of crustacean hyperglycemic hormone (CHH) neuropeptide genes across diverse crustacean species

**DOI:** 10.12688/f1000research.13732.1

**Published:** 2018-01-23

**Authors:** Wai Hoong Chang, Alvina G. Lai

**Affiliations:** 1 Nuffield Department of Medicine, University of Oxford, Oxford, Oxfordshire, OX3 7FZ, UK

**Keywords:** Crustaceans, Neuropeptides, Crustacean Hyperglycemic Hormone (CHH), Transcriptomics, Comparative genomics

## Abstract

**Background: **Recent studies on bioactive peptides have shed light on the importance of these compounds in regulating a multitude of physiological, behavioral and biological processes in animals. Specifically, the neuropeptides of the crustacean hyperglycemic hormone (CHH) superfamily is known to control a number of important functions ranging from energy metabolism, molting, osmoregulation to reproduction.

**Methods: **Given the importance of this peptide family, we employed a conservative approach utilizing extant transcriptome datasets from 112 crustacean species, which not only include important food crop species from the order Decapoda, but also from other lower order crustaceans (Branchiopoda and Copepoda), to identify putative CHH-like sequences.

**Results and conclusions: **Here we describe 413 genes that represent a collection of CHH-like peptides in Crustacea, providing an important staging point that will now facilitate the next stages of neuroendocrine research across the wider community.

## Introduction

Crustaceans and insects from the phylum Arthropoda have longstanding histories in peptide biology research, principally in areas related to the roles of peptide hormones in physiology and neuroendocrine signaling. Early discoveries have demonstrated that compounds in the crustacean nervous system were responsible for chromatophore control ^1– 3^. Four decades later, it was revealed that a compound known as the red pigment concentrating hormone functions as the first crustacean/invertebrate neuropeptide 4. Since then, multiple studies have shed light on the highly pleiotropic functions of crustacean neuropeptides implicated in the regulation of a myriad of physiological processes such as light adaptation, molt inhibition, carbohydrate metabolism, reproduction and ion transport ^5– 9^.

The crustacean hyperglycemic hormone (CHH) represents a neuropeptide superfamily that is unique to arthropods ^6, 10– 12^. This superfamily is made up of peptides containing ^∼^70 amino acids originally isolated from the X-organ-sinus-gland system of the decapod *Carcinus maenas* ^13^. Given their high degree of structural similarities, and the conservation of six cysteine residues, the molt-inhibiting hormone (MIH) and gonad-inhibiting hormone (GIH) were considered as part of this family collectively known as CHH/MIH/GIH. To date, at least 150 CHH peptides have been isolated and characterized, mainly in decapods through comparative studies on endocrinology ^5– 7, 14– 21^. Although there are reports on CHH peptides in other crustacean taxa such as *Armadillidium vulgare* (Isopoda) ^22, 23^, *Daphnia pulex* (Cladocera) ^24^ and *Daphnia magna* ^15^, investigations beyond decapods have remained scant and the sequences of CHH/MIH/GIH genes in other crustacean taxa have remained elusive.

**Figure 1.**
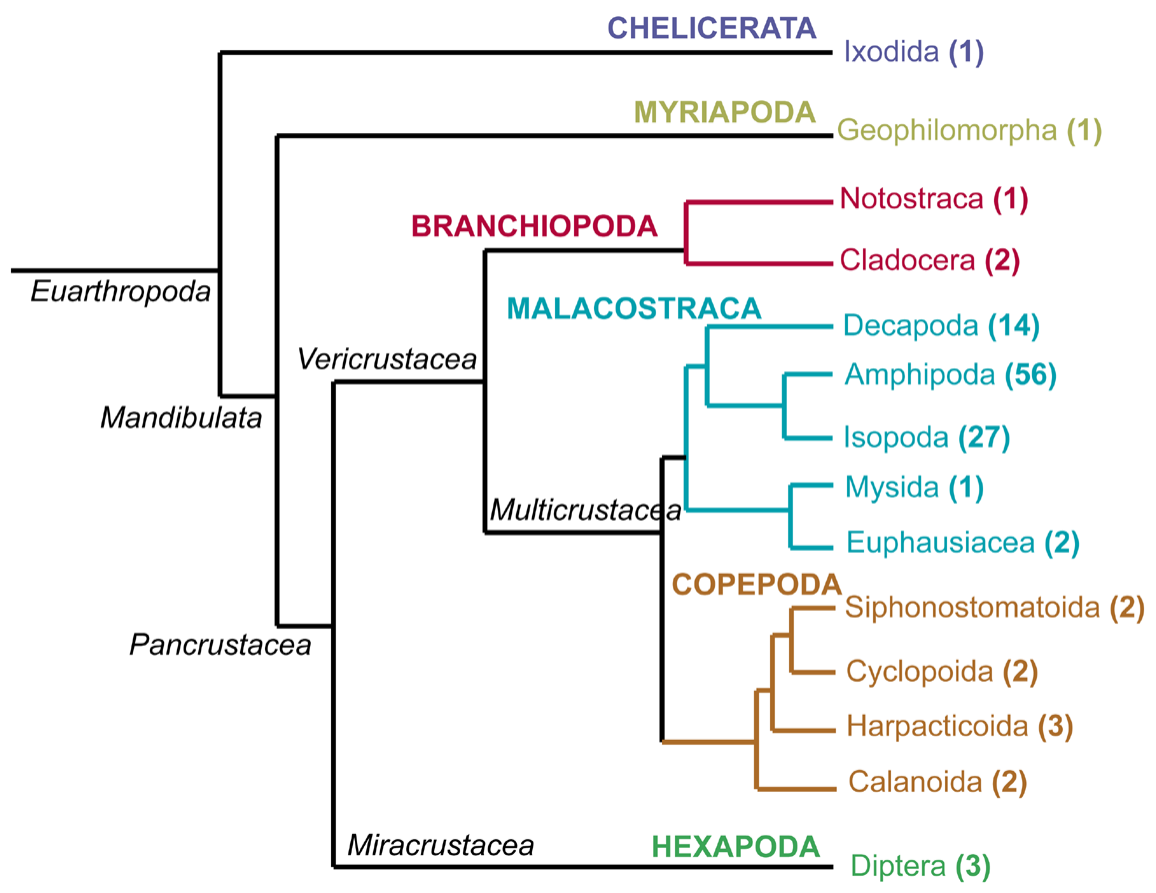
Phylogenetic relationship of Crustacea. The number of species within each taxon is denoted in parentheses.

Here, we took advantage of the growing number of high-throughput crustacean datasets on public repositories to perform transcriptome mining of the CHH/MIH/GIH superfamily. To this end, we looked at crustacean species from three Classes ( Figure 1) and annotated CHH/MIH/GIH genes. This high confidence set of genes identified using our *in silico* framework provides an important basis for understanding neuropeptide biology underpinning physiological adaptations across diverse crustacean species.

## Methods

### Transcriptome datasets and query sets

We retrieved complete transcriptome datasets for 112 crustacean species available at the time of manuscript preparation from the European Nucleotide Archive. Five non-crustacean arthropod proteomes were retrieved from Uniprot. A complete list of accessions used in this study is provided in Supplementary Table 1. We retrieved a list of query sequences used in subsequent homology searches from Uniprot and GenBank.

### Identification of CHH/MIH/GIH peptides

To identify CHH/MIH/GIH gene orthologs, we used multiple Basic Local Alignment Search Tool (BLAST)-based approaches such as BLASTp and tBLASTn with varying Blocks Substitution matrices based on a previously published workflow ^25^. The BLAST results were filtered by e-value of < 10 ^-6^, best reciprocal BLAST hits against the GenBank non-redundant (nr) database and redundant contigs having at least 95% identity were collapsed using CD-HIT. We then utilized HMMER (version 3.1) employing hidden Markov models (HMM) profiles ^26^ to scan for the presence of CHH Pfam domains ^27^ on the best reciprocal nr BLAST hits to compile a final non-redundant set of crustacean CHH/MIH/GIH orthologs. Pfam annotations, associated e-values and fasta sequences are provided in Dataset 1 ^28^ and Dataset 2 ^29^.

### Multiple sequence alignment and phylogenetic tree construction

Multiple sequence alignments of CHH protein sequences were performed using MAFFT (version 7) ^30^. Phylogenetic tree was built from the MAFFT alignment using RAxML WAG + G model to generate best-scoring maximum likelihood trees ^31^. Geneious (version 7) was used to generate multiple sequence alignment images as well as graphical representations of the Newick tree ^32^.

### Results and discussion:

**Figure 2.**
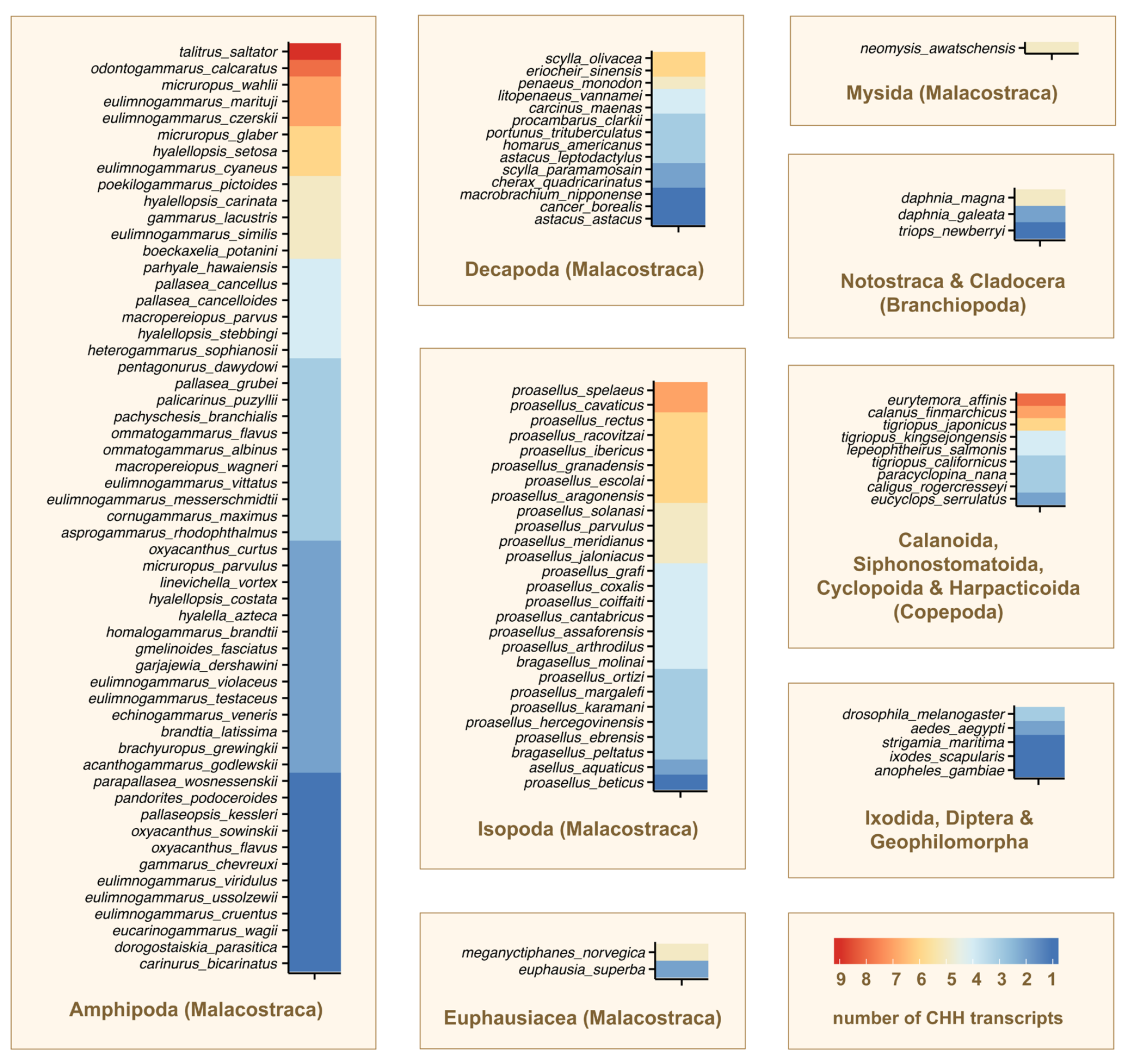
CHH/MIH/GIH genes in Crustacea. Heat maps denote the number of CHH/MIH/GIH genes identified from each crustacean species. CHH/MIH/GIH genes from five non-crustacean species within Arthropoda are also shown.

We have annotated CHH/MIH/GIH genes from 112 crustacean transcriptome datasets representing three Classes: Malacostraca (Amphipoda: 56 species, Decapoda: 14 species, Isopoda: 27 species, Euphausiacea: 2 species and Mysida: 1 species), Branchiopoda (3 species), and Copepoda (9 species) ( Supplementary Table 1). We also looked at 5 non-crustacean species from Arthropoda: Insecta (3 species), Arachnida (1 species) and Chilopoda (1 species) ( Supplementary Table 1). Using sequence and motif similarity based approaches, we have conservatively identified a total of 413 genes from these transcriptomes ( Figure 2; Dataset 1 ^28^ and Dataset 2 ^29^).

**Figure 3.**
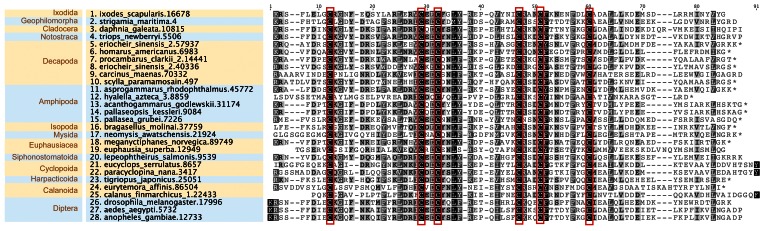
Multiple sequence alignment of representative CHH/MIH/GIH proteins from each taxon. Six conserved cysteine residues are annotated within red boxes.

**Figure 4.**
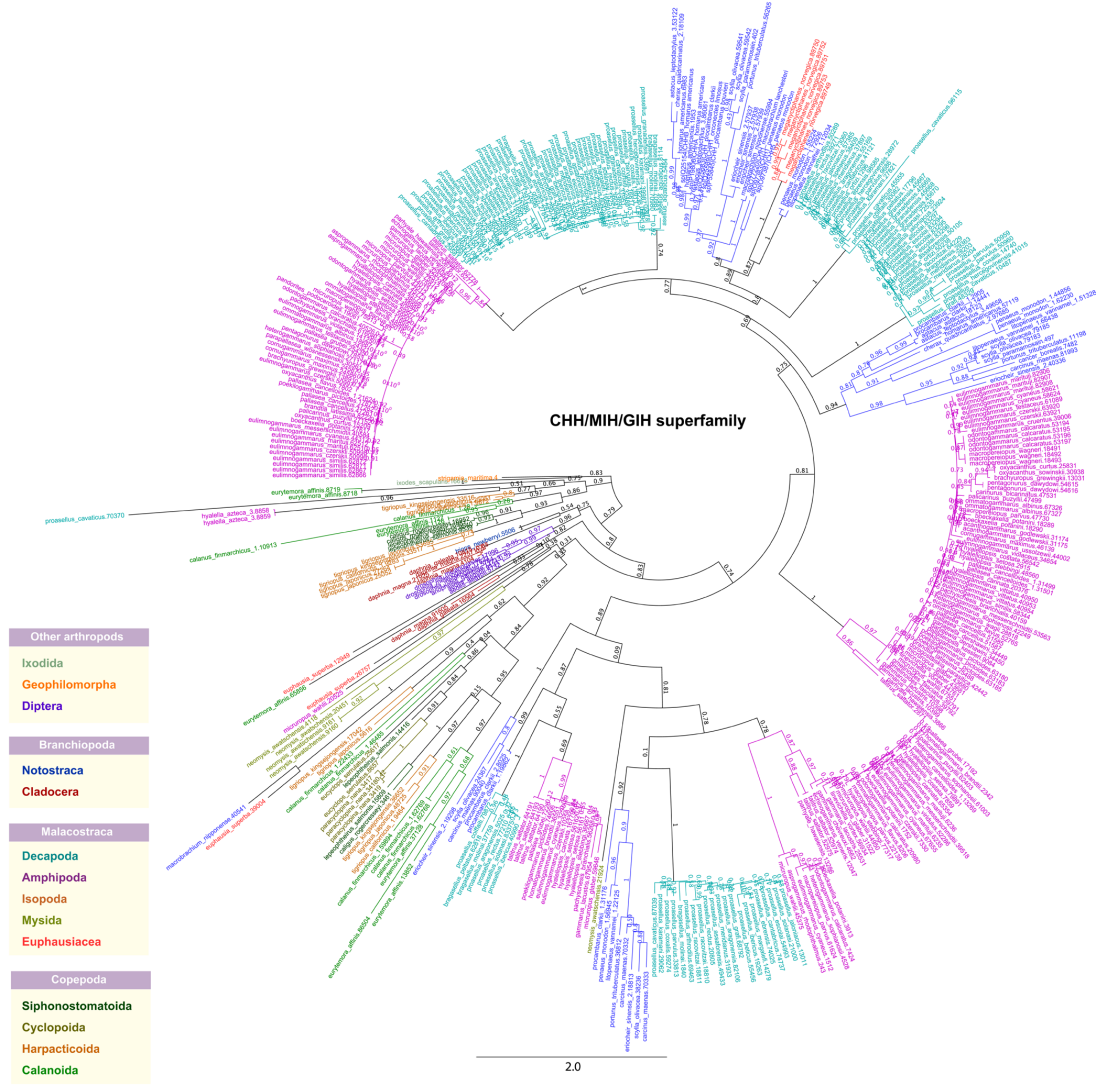
Crustacean CHH/MIH/GIH phylogeny. The tree was constructed using the maximum-likelihood method from an amino acid multiple sequence alignment. The node labels of each taxon are marked with distinctive colors denoted in the figure inset. Bootstrap support values ( *n*=1000) are denoted as branch labels.

Multiple sequence alignment analyses on representative CHH/MIH/GIH sequences revealed the presence of a conserved set of six cysteine residues ( Figure 3), likely contributing to the formation of disulfide bonds ^33^. Comparison of insect sequences from *Drosophila melanogaster, Anopheles gambiae* and *Aedes aegypti* demonstrated sequence identities of at least 46% ( Supplementary Table 2). Within crustacean taxa, a range of sequence identities were observed: Branchiopoda ( ^∼^25% to 93%), Copepoda ( ^∼^12% to 30%) and Malacostraca ( ^∼^10% to 98%) ( Supplementary Table 2). This is reflected in the phylogeny where CHH/MIH/GIH sequences from related individuals form distinct clusters ( Figure 4). It was previously reported that multiple gene duplications of CHH family peptides occurred in the decapod lineage leading to a high degree of genetic polymorphism ^15^, hence providing an explanation for our current observation. Two separate clusters of CHH genes exhibiting antagonistic patterns of expression were identified in the decapod *Metapenaeus ensis*, posited to represent an ancient gene duplication event ^34^. Although it is not possible to pinpoint the genomic loci of CHH sequences identified from this study, it is likely that paralogous copies offer mechanisms for evolving new functions through functional divergence. CHH-like genes arising from duplication of the ancestral copy are subjected to reduced selective pressure and therefore may lose their hyperglycemic activity to adopt more specialized roles ^15^. Further biochemical studies will be required to unravel the functions of the novel genes identified from this study.

Fasta file for CHH/MIH/GIH sequences in crustaceans and other arthropods Click here for additional data file. Copyright: © 2018 Chang WH and Lai AG 2018 Data associated with the article are available under the terms of the Creative Commons Zero "No rights reserved" data waiver (CC0 1.0 Public domain dedication).

List of Pfam annotated CHH/MIH/GIH genes and associated e-values in crustaceans and other arthropods Click here for additional data file. Copyright: © 2018 Chang WH and Lai AG 2018 Data associated with the article are available under the terms of the Creative Commons Zero "No rights reserved" data waiver (CC0 1.0 Public domain dedication).

## Conclusions

We have generated a high confidence list of CHH/MIH/GIH sequences from distantly related crustaceans. As a fundamental step in a broader endeavor this data is now available to the wider community to allow detail functional analyses pertinent to the next stages of neuropeptide research. Given the paucity of CHH sequences beyond decapod crustaceans, our analysis forms a promising basis for studies ranging from biochemistry to the evolution of this elusive superfamily.

## Data availability

The data referenced by this article are under copyright with the following copyright statement: Copyright: © 2018 Chang WH and Lai AG

Data associated with the article are available under the terms of the Creative Commons Zero "No rights reserved" data waiver (CC0 1.0 Public domain dedication). 

Data supporting the conclusions of this study are provided as Supplementary Material and Dataset 1 and Dataset 2.

**Dataset 1:** Fasta file for CHH/MIH/GIH sequences in crustaceans and other arthropods. 10.5256/f1000research.13732.d191194 ^28^

**Dataset 2:** List of Pfam annotated CHH/MIH/GIH genes and associated e-values in crustaceans and other arthropods. 10.5256/f1000research.13732.d191195 ^29^

## Supplementary material

**Supplementary Table 1:** List of accession numbers for species used in this study.

**Supplementary Table 2: ** Protein distance matrix (% identity) constructed from a multiple sequence alignment of CHH/MIH/GIH sequences.
