# Supplementary material for: Comparative genomic analysis of crustacean hyperglycemic hormone (CHH) neuropeptide genes across diverse crustacean species
Source: F1000Res. 2018 Jan 23;7:100. [Version 1] doi: 10.12688/f1000research.13732.1 (PMC6178914; doi:10.12688/f1000research.13732.1)
Supplement: Peer review history

# Peer review: Referee response for version 1

**DOI:** 10.5256/f1000research.14919.r37870

**Published:** 2018-10-08

**Authors:** Jos H. M. Schippers

**Affiliations:** 1 Institute of Biology, RWTH Aachen University, Aachen, Germany

The manuscript presented by Chang and Lai describes a collection of CHH neuropeptide genes in over 100 crustacean species.

 I read with interest this short report and could appreciate that a majority of neuropeptide studies have been limited to decapod crustacean species previously. In addition the presence of neuropeptide transcripts in other divergent crustaceans including those from basal lineages (copepods and branchiopods) are presented.

 Like in their previous work (Chang and Lai, 2018, BMC Genomics ^1^), a broad sampling strategy was followed. Here the approach was across multiple crustacean lineages, which allows for drawing conclusions on the evolutionary trajectory of CHH genes and their potential functional role during life histories.

 Although the paper describes a single finding, it is a valuable asset, as it provides an inventory of a high-quality list of carefully curated CHH neuropeptide transcripts for future functional studies.

 The paper is well-written and concise, and definitely fits with the scope and eligibility of the *F1000Research* guidelines.  

 As a minor comment: it would be good to describe how the tree in Figure 1 was made in the legend.

I have read this submission. I believe that I have an appropriate level of expertise to confirm that it is of an acceptable scientific standard.

## References

[1] ChangWLaiA: Mixed evolutionary origins of endogenous biomass-depolymerizing enzymes in animals. *BMC Genomics*.2018;19(1) : 10.1186/s12864-018-4861-0 10.1186/s12864-018-4861-0 29925310PMC6011409

# Peer review: Referee response for version 1

**DOI:** 10.5256/f1000research.14919.r30523

**Published:** 2018-04-20

**Authors:** Chi-Ying Lee, Jean-Yves Toullec

**Affiliations:** 1 Department of Biology, National Changhua University of Education, Changhua, Taiwan; 2 Sorbonne Universités, UPMC Université Paris 06, UMR 7144 CNRS, Equipe ABICE, Station Biologique de Roscoff, Roscoff, France

The authors collected and analyzed transcriptome datasets for 112 crustacean species (representing three Classes - Malacostraca, Branchiopoda, and Copepoda), as well as those for 5 non-crustacean (arthropod) species (Insecta, Arachnida and Chilopoda) (Supplementary Table 1; Fig. 1), retrieved from public repository.

 Collectively, 413 genes are annotated based on sequence and motif similarity as encoding peptides belonging to the so-called CHH/MIH/GIH family. The number of CHH/MIH/GIH genes identified from each crustacean species is shown as heat maps (Fig. 2). Protein sequence, annotation information, etc. are listed (Dataset 1 and 2). Between-species % identity is reported (Supplementary Table 2). Multiple sequence alignment on representative CHH/MIH/GIH sequences is presented in Fig. 3 to show the 6 conserved cysteine residues (Fig. 3). A phylogenetic tree is built based on the multiple sequence alignment data (Fig. 4).   

 The main drawback of this work is it only results in a 413-sequence inventory of CHH/MIH/GIH family peptides, but with little information (nucleotide length, amino acid sequence).

 The authors did not describe how the tree presented in Fig. 1 was built with what sorts of data. If they took a constructed tree from other source, this should be cited. There are obviously other current interpretations of a phylogenetic relationship of Crustacea, in addition to the one shown in the manuscript.

 For annotation information (Dataset 2), it should be more discerning in that peptide should be assigned as CHH, MIH/GIH, MOIH (mandibular organ-inhibiting hormone) or ITP (ion transport peptide), instead of only giving an inclusive description - Crustacean CHH/MIH/GIH neurohormone family.

 Results derived from further analysis of the sequence data (Figs, 2, 3, and 4) are not adequately discussed or lead to conclusions that have been previously established.

 For example, the only conclusion made with Fig. 3 is the 6 highly cysteine residues, a signature of the family already extensively described.  Figure 4 is left without discussion. Moreover, it is not mentioned which type of peptide (CHH, MIH/HIV, or ITP) the different clusters highlighted in the tree belong to.

 Part of the discussion (left column, p. 4) starting with “It was previously reported that multiple gene duplications of CHH family peptides…..” is not particularly relevant to the data being discussed and is again making a point already extensively described and discussed. It should also be mentioned that genomic data are more adequate than transcriptomic data when discussing gene duplication and gene copy number (Fig. 2). 

 Figure 2 is also left without discussion. In addition, data presented in this figure might be suffering from the criticism that the number of CHH/MIH/GIH genes assigned to each species is likely biased as the number will be absolutely influenced by the type of tissue used for sequencing (eyestalk ganglia, other tissue, or whole animal) and sequencing depth. Accordingly, the relevant information should be given in Supplementary Table 1 and comparison should only be made among species with the same sequenced tissue type (e.g., eyestalk ganglia) and comparable sequencing depth. 

 Overall, while the methods employed are largely (but not entirely) appropriate for the intended analysis, the goal of the study is not clearly set and this work only produces a sequence inventory without novel finding or solid discussion. Additional analyses expected to yield novel finding should be added to the manuscript. For example, with a vast amount of sequence information that could be extracted from a set of 413 peptides from animals encompassing 3 Classes, would it not be possible to uncover some function-defining sequence characteristics or motif? This piece of information would be useful for functional analysis, which the authors thought should be an important aspect of the next stage of research.

We have read this submission. We believe that we have an appropriate level of expertise to confirm that it is of an acceptable scientific standard, however we have significant reservations, as outlined above.

# Peer review: Referee response for version 1

**DOI:** 10.5256/f1000research.14919.r31165

**Published:** 2018-02-26

**Authors:** Abigail Elizur, Tuan V. Nguyen

**Affiliations:** 1 Genecology Research Center, Faculty of Science, Health, Engineering and Education, University of the Sunshine Coast, Sippy Downs, Qld, Australia

The current study is an interesting “ *in silico*” approach that mine for CHH/MIH/GIH neuropeptide in a wide range of crustacean. Introduction is updated and concise. However, there are some minor points that will need to be addressed to provide clarity information for readers. Materials and methods is sound and appropriate, although I raise some particular suggestions to further expand the quality of the MS. Results and discussions are overall good. One major point that needs to be fixed is the phylogenetics tree, whereas the color code system does not correlate between legend and figure.

 In addition, I have specific comments, (the fact that it does not appear as numbered pages and lines makes it a bit awkward, but see below):

 Page 2: …known as CHH/MIH/GIH- There are also Mandibular Inhibiting hormone (MOIH) and also ITP (Ion Transport Protein) to be part of the super family. See Ohira (2016) ^1^.

 …from three Classes - Please elaborate on this, are these branchiopoda,. malacostraca and copepoda?

 (Figure 1) – How was Figure 1 generated ? Is this a phylogenetics based tree built on mitochondrial genome or it is just an illustration?

 Genebank nr database… What about TSA (Transcriptome Shotgun Assembly) database? As that will increase the chance that the algorithm will discover more CHH/MIH/GIH? Since you mention in the introduction "Here, we took advantage of the growing number of high-throughput crustacean datasets on public repositories to perform transcriptome mining of the CHH/MIH/GIH superfamily." Adopting the TSA database will be better suited for the MS.

 …and redundant contigs… Why 95%?, let's assume that CHH prepropeptide can be roughly more than 120aa. For instance, two distinct CHH in Homarus americanus CHH-A (P19806) and CHH-B (Q25154) have 127/134 aa that are similar (~95%). Will they be collapsed with CD-HIT?

 Page 4

 Within crustacean taxa… Similarity was pretty high in Branchiopoda and Malacostraca (93% & 98%), but significantly lower in Copepoda. Can you propose an explanation for that?

 It was previously reported that… In light of this, Veenstra (2016) ^2^ has some very intensive mining of CHH in multiple decapod species. Why not provide a section that compares between the number of CHH/MIH/GIH predicted from your dataset with the above study?

 Figure 4 - Color coding system is misleading, for instance Decapoda was assigned as teal in legends, but actually blue in the phylogenetics tree.

We have read this submission. We believe that we have an appropriate level of expertise to confirm that it is of an acceptable scientific standard, however we have significant reservations, as outlined above.

## References

[1] OhiraT: Crustacean Hyperglycemic Hormone.2016; 10.1016/B978-0-12-801028-0.00053-2 403-1 10.1016/B978-0-12-801028-0.00053-2

[2] VeenstraJA: Similarities between decapod and insect neuropeptidomes. *PeerJ*.2016;4: 10.7717/peerj.2043 e2043 10.7717/peerj.2043 27257538PMC4888303
